# Operations upon Arteries

**Published:** 1909-04-17

**Authors:** 


### Surgery.

### OPERATIONS UPON ARTERIES.

Within the past few years the attention of surgeons generally, and particularly of those in America, has been directed to the subject of operations upon arteries, and the result has been such an improvement in technique that other procedures besides mere ligature are at any rate possible, even if they are not always advisable. Like all new work, this successful result has been a gradual evolution, the outcome of empiricism. The best known of the earlier methods advocated for the end-to-end union of arteries was Murphy's. In this the proximal end of the cut artery was invaginated into the distal, and the edges of the distal end united to the two outer coats as they lay in apposition. Experience showed, however, that the projecting proximal end allowed fibrin ferment to escape into the circulation, and thrombosis followed, a result which rendered the operation impracticable. It was next proposed to unite the divided ends by sutures which did not penetrate the lumen of the vessel. With this object sutures were used which passed only through the outer and middle coats, but it was found that this more often than not gave rise to a dissecting aneurysm, the stream of blood passing down underneath the ununited inner coat and stripping it up from the tunica media. But recently it has been shown that it is possible to unite an artery safely and securely by means of an accurately adjusted continuous mattress suture which passes through all the coats; the edges are slightly everted so that when the suture is tightened up and tied no part of it projects into the lumen of the vessel, and leakage does not occur. A second continuous suture over all, uniting the edges, may be inserted for greater security, but this is not absolutely necessary. This operation has now been performed for wounds of large arterial trunks, for the removal of an embolus, and for reversing the circulation in the limb in cases of incipient gangrene. But it will be readily understood that the indications for its use are somewhat circumscribed.

A still further advance in the surgery of the arterial system has been made in the operation of endo-aneurysmorrhaphy, which has been devised by Dr. Matas, of New Orleans, for the treatment of aneurysms. Strictly speaking, he has described two separate methods of operating on an aneurysm from the interior of the sac. The first he calls the "restorative." In this, which is only applicable to sacculated aneurysms, the connection of the aneurysm with the lumen of the vessel is obliterated, while the continuity of the vessel itself is not interfered with. The second he calls the "obliterative"; and he reserves this for fusiform aneurysms, in which the whole circumference of the vessel is involved in the pathological process.

To ensure a successful result every detail of the technique must be carefully considered. The first, and perhaps most essential, point is to compress all the arterial trunks communicating with the aneurysm, so as to secure what he calls an ischæmic operation field. This is done by clamping the vessels with special clamps whose blades are covered with indiarubber, like gastro-jejunostomy clamps, and are approximated with a screw. The best pattern is that known as Crile's. It is necessary to secure in this way not only the parent vessel as it leads into and away from the aneurysm, but also any tributary vessels which pour their stream into the aneurysm at some intermediate point. Otherwise when the sac is opened troublesome hæmorrhage is likely to obscure the field of operation.

Secondly, the connections of the aneurysm should not be needlessly disturbed, but an incision made down on to it, important structures being alone retracted. The sac of the aneurysm is then opened by a straight incision parallel to the long axis of the vessel, or, if more exposure is desired, two elliptical incisions may be made in the sac, and the intervening portion of the sac wall removed, an oval window thus being formed.

When the interior of the aneurysm is exposed the subsequent steps depend upon the conditions found. In the case of a sacculated aneurysm there is only one opening between the vessel and the sac. This can be closed by sutures in such a way as to shut off the aneurysm completely without interfering with the lumen of the vessel. The method is as follows: The sutures used should be of chromicised gut; these are sufficiently resistant to secure permanent approximation of the edges, but are eventually absorbed. They are passed with a fine, fully-curved, rounded needle on a needle holder, starting from a point well outside the edges of the aperture and being brought out at its margin. It is better to use interrupted sutures, and they should be about an eighth of an inch from each other.

In dealing with a fusiform aneurysm it is impossible to retain a pervious vessel, and in this case the sutures are passed as before, but with this difference, that they are not brought at the margin of the aperture, but pick up the floor of the vessel as it enters the sac; so that when the sutures are tied the orifice of the vessel is obliterated. Every vessel entering the sac must be treated in this way.

The subsequent measures are the same in either case. The sac wall may be doubled on itself or its outer wall may be cut away entirely. Whichever of these alternatives is adopted, deep sutures are passed by which the remains of the sac are sutured to the overlying skin.

Up to the present time, roughly, 100 of these operations have been performed, mostly in America, with a mortality of 9 per cent.

Dr. Matas, to whose ingenuity we owe these procedures, is strongly convinced that they are in advance of the methods hitherto practised. But until one has further experience of the success and applicability of the method, it is not easy to share his enthusiasm, however much one may admire his courage and ability. The technique is complicated, and needs special experience, and the percentage of successful cases is therefore not likely to be high, except in the hands of a more than usually skilled operator.

Moreover, in small aneurysms in the distal parts of the limbs, excision of the sac after ligature of the parent stem is a highly satisfactory proceeding, and we cannot see that Dr. Matas's obliterative method is any advance on this. The restorative operation has, it is true, the advantage of leaving the lumen of the vessel patent; but then the collateral circulation is almost always opened up with astonishing rapidity after ligature of a vessel, so that here the advantage is more apparent than real. But that there is a future for the operation there can be no reasonable room for doubt, though its field of usefulness will probably be narrow—*i.e.*, for sacculated aneurysms of large trunks, such as the aorta—in which ligature of the proximal stem is either impossible or inadvisable.

